# MiR-19a, miR-122 and miR-223 are differentially regulated by hepatitis B virus X protein and involve in cell proliferation in hepatoma cells

**DOI:** 10.1186/s12967-016-0888-7

**Published:** 2016-05-05

**Authors:** Guifang Yu, Xuezhu Chen, Shudi Chen, Weipeng Ye, Kailian Hou, Min Liang

**Affiliations:** Department of Oncology, The Fifth Affiliated Hospital of Guangzhou Medical University, No.621, Gangwan Road, Huangpu District, Guangzhou, 510700 China

**Keywords:** Hepatitis B virus X protein, microRNAs, Hepatocellular carcinoma, HepG2, PTEN, Cyclin G1, c-myc

## Abstract

**Background:**

Hepatitis B virus (HBV) X protein (HBx) is a type of oncogenic protein involved in the progression of hepatocellular carcinoma (HCC) via interacting with host genes. Dysregulation of microRNAs (miRNAs) has been observed in HCC. This study aimed to investigate the role of HBx protein in the regulation of miR-19a, miR-122 and miR-223, and examine if these miRNAs involve in progression of malignant hepatocytes.

**Methods:**

Quantitative real time PCR (qRT-PCR) was used to measure the expression of miR-19a, miR-122 and miR-223 in patient samples and in HepG2 cells transfected with HBx or 1.3 fold HBV genome and also in HepG2.2.15 cells, which stably produces HBV. Their target mRNAs and proteins-PTEN, cyclin G1 and c-myc were measured by qRT-PCR and western blot, respectively. The effect of miR-19a, miR-122 and miR-223, and their respective target genes, on cell proliferation was analyzed using 5-ethynyl-2-deoxyuridine incorporation and MTT assay.

**Results:**

MiR-19a showed an up-regulation in HBV-positive HCC patients compared to healthy controls and HBV-negative HCC patients, while miR-122 and miR-223 showed a down-regulation compared to healthy controls, and miR-122 in HBV-positive HCC patients was also down-regulated when compared to HBV-negative HCC patients. MiR-19a was found to be up-regulated in HepG2 cells transfected with HBx or 1.3 fold HBV genome, but down-regulated in HepG2.2.15 cells. MiR-122 and miR-223 were down-regulated in HBx or 1.3 fold HBV transfected HepG2 cells as well as in HepG2.2.15 cell. Their target mRNAs and corresponding proteins-PTEN was down-regulated, while cyclin G1 and c-myc were found to be up-regulated. Modulated expression of miR-19a, miR-122 and miR-223 enhanced cell proliferation of HBx-transfected HepG2 cells, and rescue experiment further showed that their target genes-PTEN, cyclin G1and c-myc involved in cell proliferation of HBx-transfected HepG2 cells.

**Conclusions:**

The expression of miR-19a, miR-122 and miR-223 were differentially regulated by HBx protein, the differential expression of miR-19a, miR-122 and miR-223 plays an important role in cell proliferation of HCC. This study provides new insight into understanding how HBx protein interacts with miRNAs and subsequently regulates host function.

## Background

Hepatocellular carcinoma (HCC) is one of the most common forms of human cancer in the world and the leading cause of cancer-related death worldwide [1]. In China, chronic Hepatitis B Virus (HBV) infection is a major risk factor of HCC and liver cirrhosis [2]. Recent studies showed that HBV X protein (HBx) plays an important role in the pathogenic mechanism of HBV-associated HCC [3]. HBx is a multifunctional oncogenic protein, which interacts with various transcription factors (e.g., Oct-1, ATF-2, CREB) and modulates numerous signaling pathways (e.g., Src, AP-1, Ras, PI3 K/Akt, Jak/STAT and Wnt) [4, 5]. Recent studies also showed that HBx may increase telomerase activity to increase the lifespan of hepatocytes and subsequently transform them to malignancies [6]. Therefore, HBx causes sustained changes in expression of cellular genes, which may result in enhanced hepatocytes growth and proliferation and lead to HCC.

MicroRNAs (miRNAs) are a class of endogenous, non-coding, short RNAs, and negatively regulate the target genes via binding to the 3′ untranslated regions. MiRNAs have been shown to involve various biological processes, including cell proliferation, apoptosis, and differentiation [7]; and dysregulation of miRNAs have been associated with various types of cancers in human [8]. MiRNAs have been shown to function as oncogenes or tumor suppressor genes during tumor development and progression [9]. Differential expression of miRNAs in HCC patients compared to healthy subjects have been studied. MiR-224, miR-19a and miR-27a are upregulated in HCC; while miR-122, miR-223 and miR-101 are down-regulated in HCC [10]. Overexpression of miR-122 and miR-223 directly results in the down-regulation of cyclin G1 and c-myc, respectively [11, 12].

Increasing efforts have been made in deciphering the interaction between HBx and miRNAs. One study showed that HBx negatively regulates let-7a, and down-regulation of let-7a has been shown to increase cell proliferation in HepG2 and SNU-182 cells [13]. MiR-27a is found to be up-regulated in HBV-positive HCC patients, and the up-regulation of miR-27a results in enhanced cell proliferation, migration, and invasion [14]. MiR-101 is negatively regulated by HBx and induces aberrant DNA methylation by targeting DNA methyl transferase 3A [15]. Although miRNAs have been shown to be regulated by HBV, how dysregulation of miRNAs influences liver cancer development and progression remains largely unknown.

In the present study, we first investigated differential expression of miR-19a, miR-122 and miR-223 in HBx or 1.3 fold HBV transfected HepG2 cells and in HepG2.2.15 cells, as well as in HBV-negative HCC patients and HBV-positive HCC patient samples. The role of HBx in modulation of expressions of these miRNAs in malignant hepatocytes was further validated by HBx gene knock down study. Next, we also examined whether differential expression of miR-19a, miR-122 and miR-223 is associated with the changes in expression levels of its target mRNAs and corresponding proteins (PTEN, cyclin G1 and c-myc of miR-19a, miR-122 and miR-223, respectively) [11, 12, 16]. Finally, we determined roles of miR-19a, miR-122, miR-223 and their target genes, in cell proliferation using EdU incorporation and MTT assay in HBx-transfected HepG2 cells.

## Methods

### Patient samples

Forty-three clinical HCC patients tested for HBV (genotype C)-positive result and 30 clinical HCC patients tested for HBV-negative result were recruited in this study. For the healthy controls, 43 age and sex matched heathy individuals were recruited in this study, and these healthy control were subjected to blood test to rule out the HBV infection. The blood samples were taken from all these subjects for the quantitative real time PCR (qRT-PCR) analysis of miR-19a, miR-122 and miR-223 expression. This study was approved by the Ethics Review Committees of the Fifth Affiliated Hospital of Guangzhou Medical University, and informed consent was obtained from all patients.

### Cell culture

The cells of HepG2 (American Type Culture Collection, USA) and HepG2.2.15 (Shanghai Second Military Medical University), which constitutively replicated HBV, were cultured in Dulbecco’s modified Eagle medium (DMEM) with 10 % fetal bovine serum (Life Technologies Inc, Gaithersburg, MD, USA). Cell were incubated at 37 °C in a humidified atmosphere with 5 % CO_2_.

### Plasmids and RNA oligonucleotides

The expression vector of pcDNA3.1-HBx was constructed by inserting HBx DNA fragments into pcDNA3.1 vector [17]. The 1.3 fold HBV genome (genotype C) fragment was amplified from pGEM-HBV1.3, and the amplified 1.3 fold HBV genome fragments was cloned into pcDNA3.1 plasmid for the construction of pcDNA3.1-HBV plasmid [18]. The expression vectors of pcDNA3.1-PTEN, pcDNA3.1-cylcin G1, and pcDNA3.1-c-myc were purchased from Genepharma (Shanghai, China). HBx-siRNA, PTEN-siRNA, cyclin G1-siRNA and c-myc-siRNA was used to produce small interfering RNAs targeting HBx mRNA, PTEN mRNA, and cyclin G1 mRNA and c-myc mRNA respectively; miR-19a inhibitor was used to reduce the expression level of miR-19a, miR-122 and miR-223 mimics were used to increase the expression levels of miR-122 and miR-223, respectively; siRNA duplexes and miRNAs with non-specific sequences were designed as negative control (NC) (Ribobio, Guangzhou, China).

### Cell transfection

HepG2 cells were seeded for 24 h, and transfected with Lipofecatmine 2000 and pcDNA3.1-HBx (2 µg) or pcDNA3.1-HBV (2 µg) or empty vector, pcDNA3.1 (2 µg) (Invitrogen, CA, USA) according to the manufacturer’s instruction. In the HBx and HBV plasmid transfection experiments, cells were used for RNA extraction 48 h post-transfection. In the HBx-siRNA experiment, HBx-siRNA or its negative control was co-transfected with pcDNA3.1-HBx or pcDNA3.1-HBV plasmid in HepG2 cells, and HBx-siRNA or its negative control was transfected in HepG2.2.15 cells; RNA were extracted 24, 48, and 72 h post-transfection. For the miR-19a inhibitor, miR-122 and miR-223 mimics experiments, miR-19a inhibitor, miR-122 and miR-223 mimics or their negative controls were co-transfected with pcDNA3.1-HBx in HepG2 cells. For the PTEN overexpression, cyclin G1 and—cymc silence experiment, pcDNA3.1-PTEN, c-myc-siRNA or cyclin G1-siRNA, or their negative controls were co-transfected with pcDNA3.1-HBx in HepG2 cells; for the rescue experiment, miR-19a inhibitor + PTEN-siRNA, miR-223 mimics + pcDNA3.1-cyclin G1, or miR-122 mimics + pcDNA3.1-c-ymc were co-transfected with pcDNA3.1-HBx in HepG2 cells.

### RNA extraction, reverse transcription PCR and qRT-PCR analysis

Total RNA and miRNA fractions were isolated from blood samples, HepG2 cells and HepG2.2.15 cells using TRIzol reagent (Invitrogen, CA, USA). MiRNA extraction was performed using the miRNA Extraction Kit (Tiangen, Beijing, China). QRT-PCR was performed with SYBR Premix Ex Taq (TaKaRa). GAPDH was used as an internal control for mRNA, and RNU6B was used as the miRNA reference. The relative quantitative analysis of data were performed using 7000 system SDS software v1.2.3 (Applied Biosystems). The relative quantitation of target gene expression was obtained using the comparative ΔΔC_T_ method.

### Western blot analysis

After 48 h of transfection, proteins were extracted from HepG2 cells and HepG2.2.15 cells, and their concentrations were measured. Western blot analysis was performed using anti-PTEN (1:500, Abcam Cambridge, MA, USA); anti-cyclin G1 (1: 500, Abcam Cambridge, MA, USA); anti-c-myc (1:500, Abcam Cambridge, MA, USA); anti-beta-catenin (1:2000, Abcam Cambridge, MA, USA), and beta-actin was used as loading control. Protein bands were quantified using Dentiometric scanner (Bio-Rad-GS-800, USA).

### Cell proliferation assay

After 24 h transfection, the 5-ethynyl-2-deoxyuridine (EdU) incorporation assay was performed using the Cell-Light EdU imaging detecting kit according to the manufacturer’s instructions (Ribobio, Guangzhou, China) to assess cell proliferation. MTT assays were also used to assess cell proliferation from 24 h until 72 h post transfection. The MTT assay was performed as follows: 20 μL of MTT (5 mg/mL) was added to each well and the plates were incubated at 37 °C for 4 h. The MTT medium mixture was then removed and 150 μL of dimethyl sulfoxide was added to each well. The absorbance was measured at 570 nm using a multi-well spectrophotometer.

### Statistical analysis

All statistical analysis was carried out using GraphPad Prism version 6 (GraphPad Prism version 6.0, Inc. California, USA). The differences among groups were analyzed by one-way ANOVA followed by Bonferroni’s multiple comparison tests or *t* test, as appropriate. All data are expressed as mean ± SEM. Differences were considered significant when *P* < 0.05.

## Results

### Expression of miRNAs among the HBV-negative HCC patients, HBV-positive HCC patients and healthy controls

**Fig. 1:**
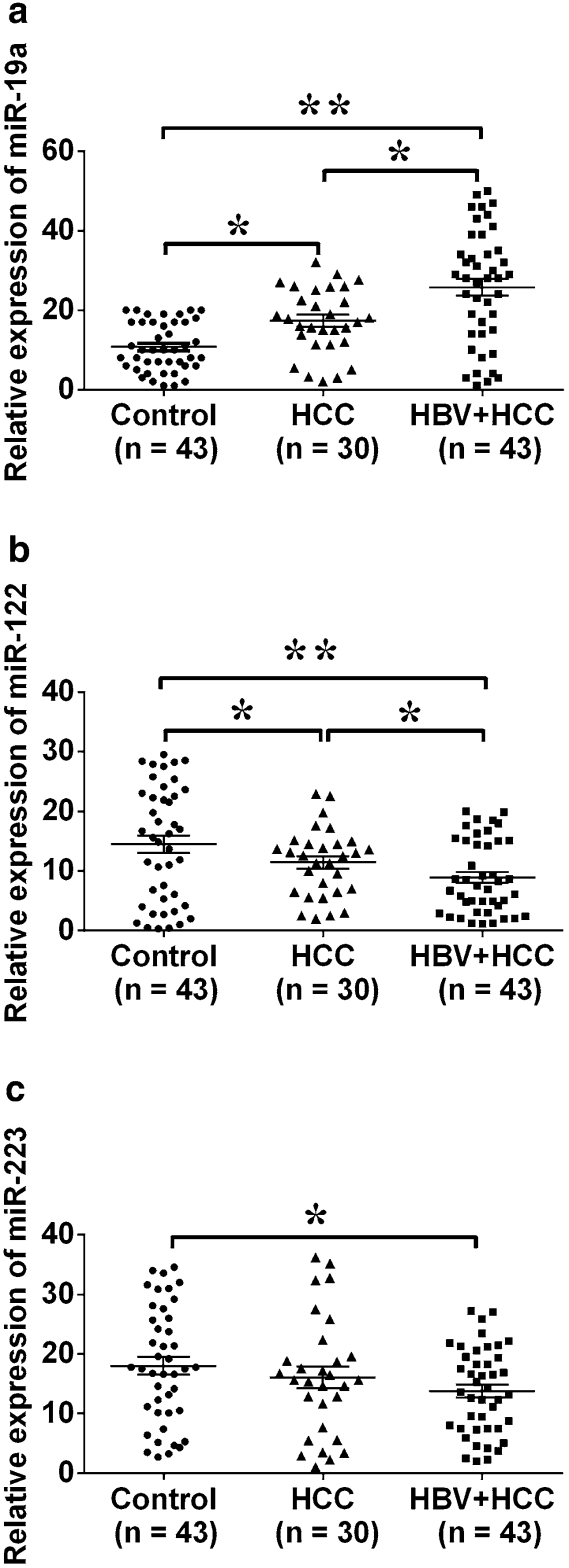
Expression levels of miR-19a, miR-122, and miR-223 in healthy subjects and patients samples. qRT-PCR analysis of **a** miR-19a, **b** miR-122, and **c** miR-223 from blood serum samples in healthy control, HBV-negative HCC patients and HCC patients with HBV infection. *HCC* hepatocellular carcinoma, *HBV* hepatitis B virus. Data represents the mean ± SEM, n = 3, *P < 0.05; **P < 0.01 (One-way ANOVA followed by Bonferroni test)

In our study, all the HBV-positive HCC patients had a genotype C HBV positive results from the blood test. The expression levels of miR-19a, miR-122 and miR-223 in blood serum from HBV-negative HCC patients, HBV-positive HCC patients and age and sex matched healthy controls were analyzed by qRT-PCR. The expression level of miR-19a was higher in the blood serum from HBV-negative HCC patients and HBV-positive HCC patients than that in healthy controls (Fig. 1a, n = 43, *P* < 0.05), and the expression level of miR-122 was lower in the blood serum from HBV-negative HCC patients and HBV-positive HCC patients compared to healthy controls; while the up-regulation of miR-223 was only found in HBV-positive HCC patients but not in HBV-negative patients when compared to healthy controls (Fig. 1b, c, n = 30 or 43, *P* < 0.05). We further compared the expression levels of these miRs between HBV-negative HCC patients and HBV-positive HCC patients, and our results showed that HBV-positive HCC patients had a higher expression level of miR-19a and a lower expression level of miR-122 than HBV-negative patients (Fig. 1a, b, n = 30 and 43, *P* < 0.05); while there was no significant differences in miR-223 expression level between HBV-negative HCC patients and HBV-positive HCC patients (Fig. 1c, n = 30 or 43, *P* > 0.05).

### Differential expression of miR-19a, miR-122, and miR-223 in HepG2 cells transfected with HBx or HBV and in HepG2.2.15 cells

**Fig. 2:**
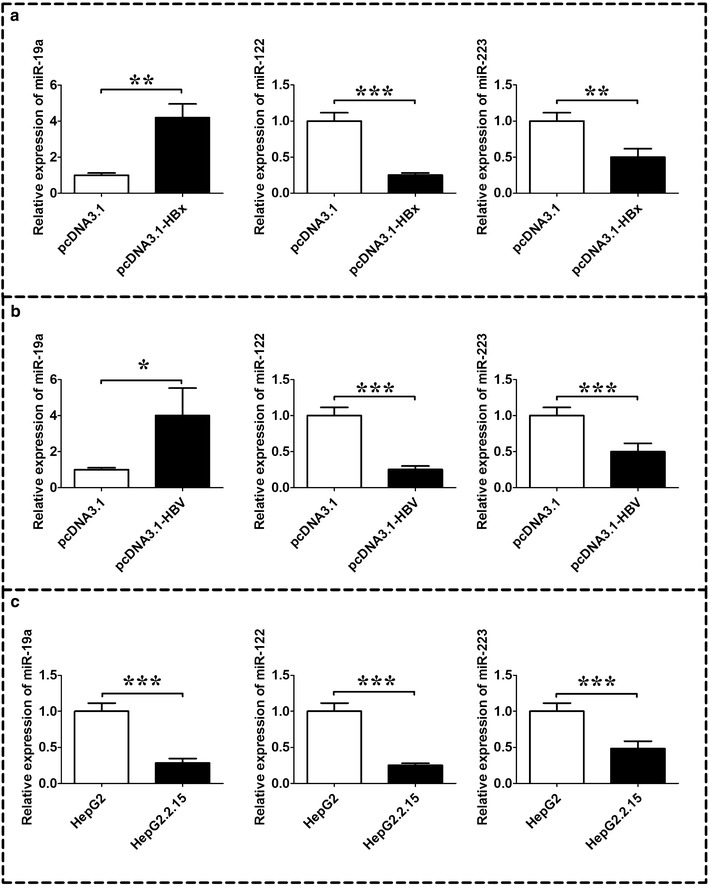
Expression levels of miR-19a, miR-122, and miR-223 in HepG2 cells transfected with HBx or HBV and in HepG2.2.15 cells. **a** qRT-PCR analysis of miR-19a, miR-122, and miR-223 in HepG2 cells transfected with HBx expressing plasmid pcDNA3.1-HBx or its control vector, pcDNA3.1; **b** qRT-PCR analysis of miR-19a, miR-122, and miR-223 in HepG2 cells transfected with 1.3 fold HBV expressing plasmid pcDNA3.1-HBV or its control vector, pcDNA3.1; **c** qRT-PCR analysis of miR-19a, miR-122, and miR-223 in HepG2.2.15 cells and its control, HepG2 cell lines. *HBV* hepatitis B virus, *HBx* HBV X protein. Data represents the mean ± SEM, n = 3, *P < 0.05; **P < 0.01; ***P < 0.001 (unpaired t-test)

QRT-PCR confirmed that expression levels of HBx was higher in HBx-transfected or 1.3 fold HBV transfected HepG2 cells compared to their respective controls (data not shown). QRT-PCR analysis showed that miR-19a was up-regulated, and miR-122 and miR-223 were down-regulated in HBx-transfected HepG2 cells compared to the HepG2 cells transfected with control vector, pcDNA3.1 (Fig. 2a, n = 3, *P* < 0.05). Transfection of HepG2 cells with 1.3 fold HBV genome have showed similar results that miR-19a was up-regulated, and miR-122 and miR-223 were down-regulated in 1.3 fold HBV-transfected HepG2 cells compared to the HepG2 cells transfected with control vector, pcDNA-3.1 (Fig. 2b, n = 3, *P* < 0.05). We also measured the expression of these miRNAs in a stable HBV producing cells, HepG2.2.15, and we found that all the miRNAs i.e. miR-19a, miR-122 and miR-223 were down-regulated in the Hepg2.2.5 cells compared to its control, HepG2 cells (Fig. 2c, n = 3, *P* < 0.05).

### Expression levels of PTEN, cyclin G1 and c-myc in HepG2 cells transfected with HBx or 1.3 fold HBV and in HepG2.2.15 cells

**Fig. 3:**
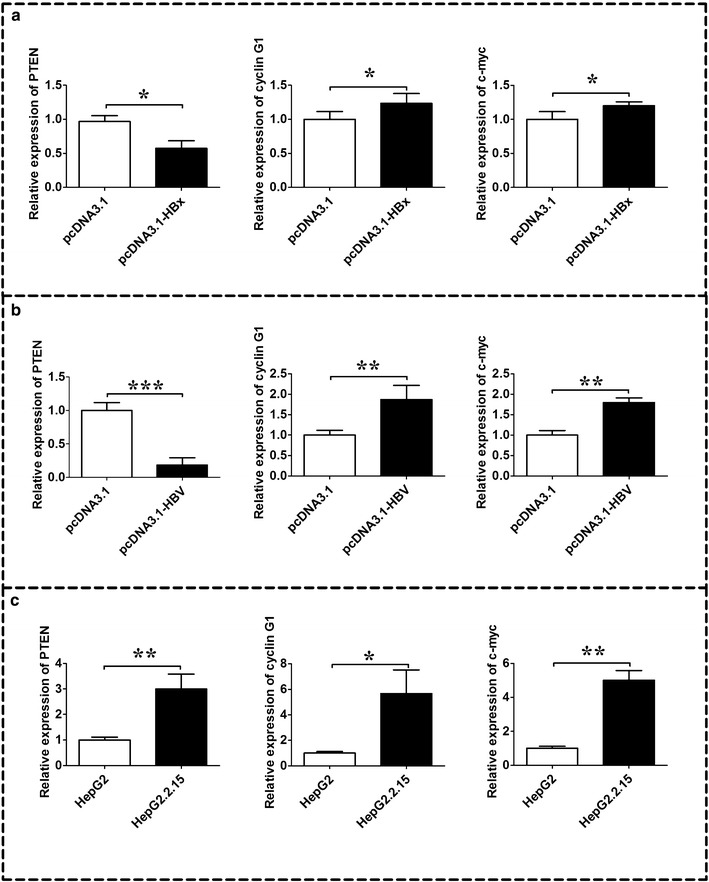
mRNA expression levels of PTEN, cyclin G1 and c-myc in HepG2 cell lines transfected with HBx or HBV and in HepG2.2.15 cells. **a** mRNA expression levels of PTEN, cyclin G1 and c-myc in HepG2 cells transfected with HBx expression plasmid pcDNA3.1-HBx or its control vector, pcDNA3.1; **b** mRNA expression levels of PTEN, cyclin G1 and c-myc in HepG2 cells transfected with 1.3 fold HBV expression plasmid pcDNA3.1-HBV or its control vector, pcDNA3.1; **c** mRNA expression levels of PTEN, cyclin G1 and c-myc in HepG2.2.15 cells and its control, HepG2 cells. HBV hepatitis B virus, *HBx* HBV X protein. Data represents the mean ± SEM, n = 3, *P < 0.05; **P < 0.01; ***P < 0.001 (unpaired t-test)

**Fig. 4:**
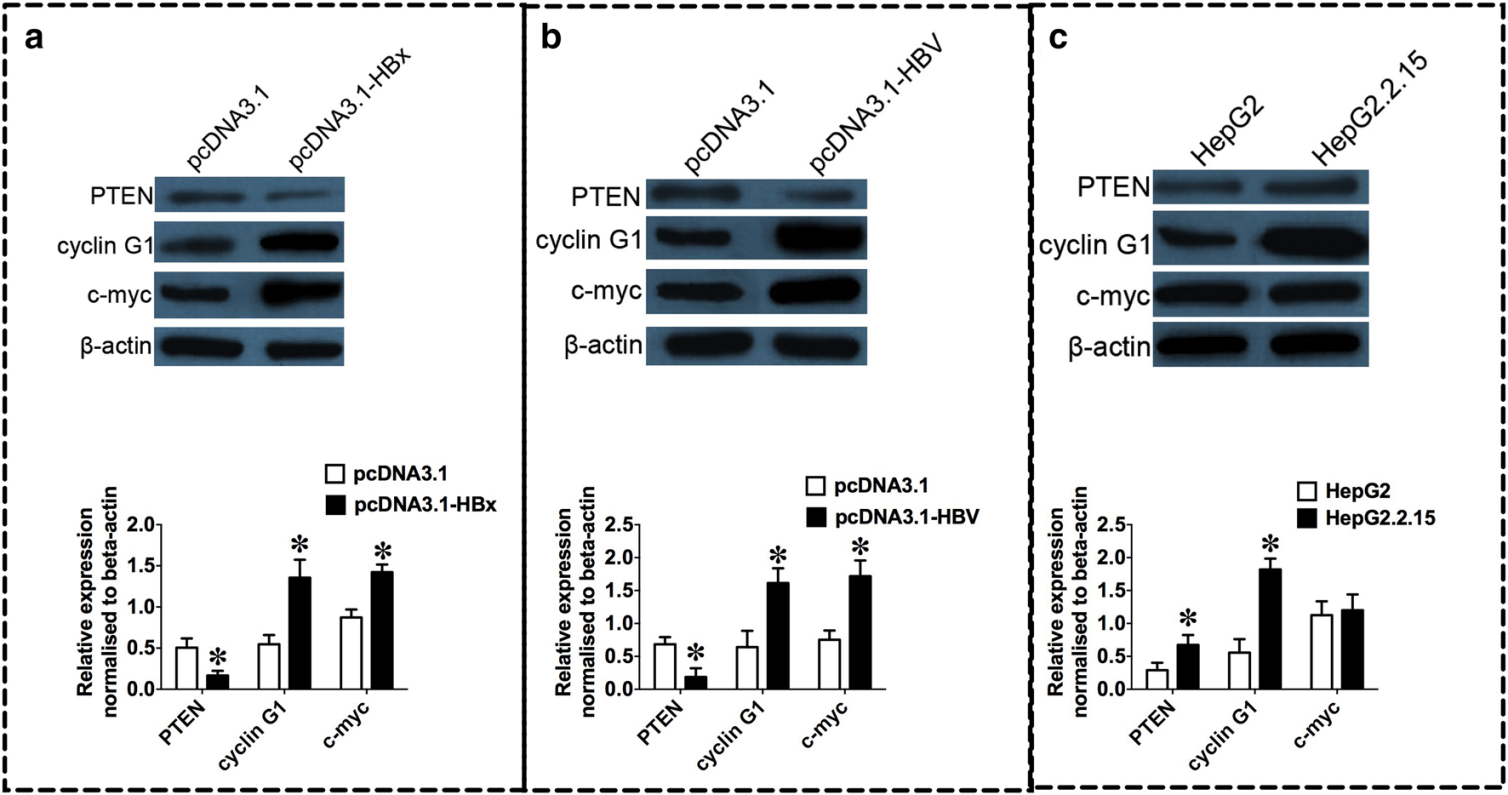
Protein expression levels of PTEN, cyclin G1 and c-myc in HepG2 cell lines transfected with HBx or 1.3 fold HBV and in HepG2.2.15 cells. **a** Western blot analysis of PTEN, cyclin G1 and c-myc in HepG2 cells transfected with HBx expression plasmid pcDNA3.1-HBx or its control vector, pcDNA3.1; **b** Western blot analysis of PTEN, cyclin G1 and c-myc in HepG2 cells transfected with 1.3 fold HBV expression plasmid pcDNA3.1-HBV or its control vector, pcDNA3.1; **c** Western blot analysis of PTEN, cycling G1 and c-myc in HepG2.2.15 cells and its control, HepG2 cells. *HBV* hepatitis B virus, *HBx* HBV X protein. Data represents the mean ± SEM, n = 3, *P < 0.05 (unpaired t-test)

QRT-PCR analysis showed that PTEN mRNA was down-regulated, and cyclin G1, c-myc mRNA were up-regulated in HepG2 cells transfected with HBx compared to HepG2 cells transfected with pcDNA3.1 (Fig. 3a, n = 3, *P* < 0.05).Transfection of HepG2 cells with 1.3 fold HBV also caused down-regulation of PTEN mRNA, and up-regulation of cyclin G1, c-myc mRNA compared to HepG2 cells transfected with pcDNA3.1 (Fig. 3b, n = 3, *P* < 0.05). In the HepG2.2.15 cells; all the mRNAs-PTEN, cyclin G1 and c-myc were up-regulated in the HepG2.2.15 cells when compared to its negative control, HepG2 cells (Fig. 3c, n = 3, *P* < 0.05). Western blot analysis further showed a decrease in PTEN protein level, and an increase in cyclin G1 and c-myc protein levels in HepG2 cells transfected with HBx or 1.3 fold HBV, and an increase in PTEN and cyclin G1 protein levels in HepG2.2.15 cells when compared to their respective controls (Fig. 4, n = 3, *P* < 0.05).

### Expression of miR-19a, miR-122, and miR-223 was differentially regulated by HBx

**Fig. 5:**
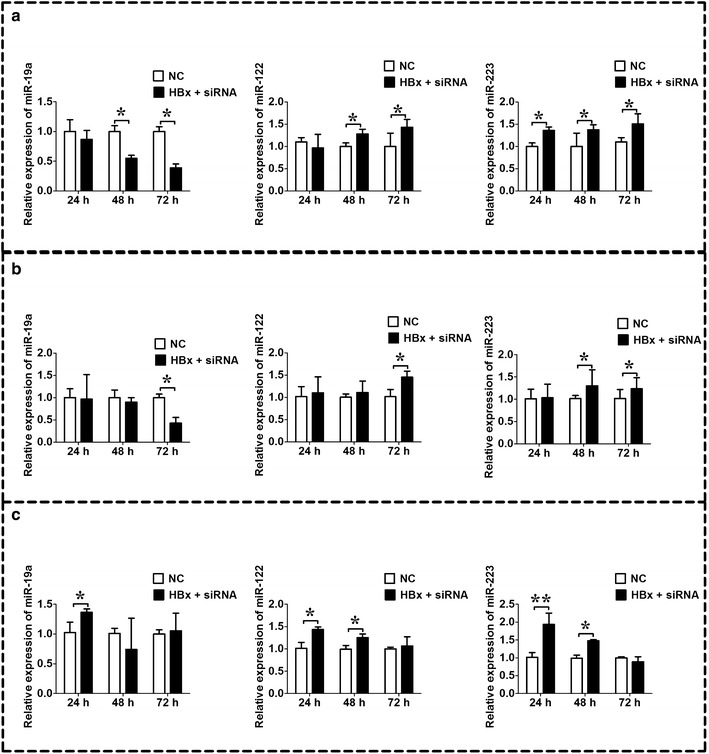
Expression levels of miR-19a, miR-122, and miR-223 after silence of HBx in HepG2 cell lines transfected with HBx or HBV and in HepG2.2.15 cells. **a** qRT-PCR analysis of miR-19a, miR-122, and miR-223 in HepG2 cells co-transfected with pcDNA3.1-HBx and HBx-siRNA or its negative control (NC); **b** qRT-PCR analysis of miR-19a, miR-122, and miR-223 in HepG2 cells co-transfected with pcDNA3.1-HBV and HBx-siRNA or its negative control (NC); **c** qRT-PCR analysis of miR-19a, miR-122, and miR-223 in HepG2.2.15 cells and its negative control, HepG2 cells. *HBV* hepatitis B virus, *HBx* HBV X protein. Data represents the mean ± SEM, n = 3, *P < 0.05; **P < 0.01 (unpaired t-test)

The knock down effect of HBx mRNA on the expression of miR-19a, miR-122, and miR-223 examined in HepG2 cells transfected with HBx plasmid; 1.3 fold HBV genome and in HepG2.2.15 cells. In HepG2 cells co-transfected with HBx plasmid and HBx-siRNA, the expression of miR-19a was lower 48 and 72 h post-transfection; the expression of miR-122 was higher 48 and 72 h post-transfection, and the expression of miR-223 was higher 24, 48 and 72 h post-transfection (Fig. 5a, n = 3, *P* < 0.05). In cells co-transfected with 1.3 fold HBV and HBx-siRNA, the expression of miR-19a was lower 72 h post-transfection; the expression of miR-122 and miR-223 was higher 72 h post-transfection, and the expression of miR-122 was also higher 48 and 72 h post-transfection (Fig. 5b, n = 3, *P* < 0.05). However, the expression levels of miR-19a was higher in HepG2.2.15 cells transfected with HBx-siRNA 24 h post-transfection; and the expression levels of miR-122 and miR-223 were also higher in HepG2.2.15 cells transfected with HBx-siRNA 24 h and 48 h post-transfection (Fig. 5c, n = 3, *P* < 0.05).

### The proliferation of HepG2 cells was enhanced by HBx

**Fig. 6:**
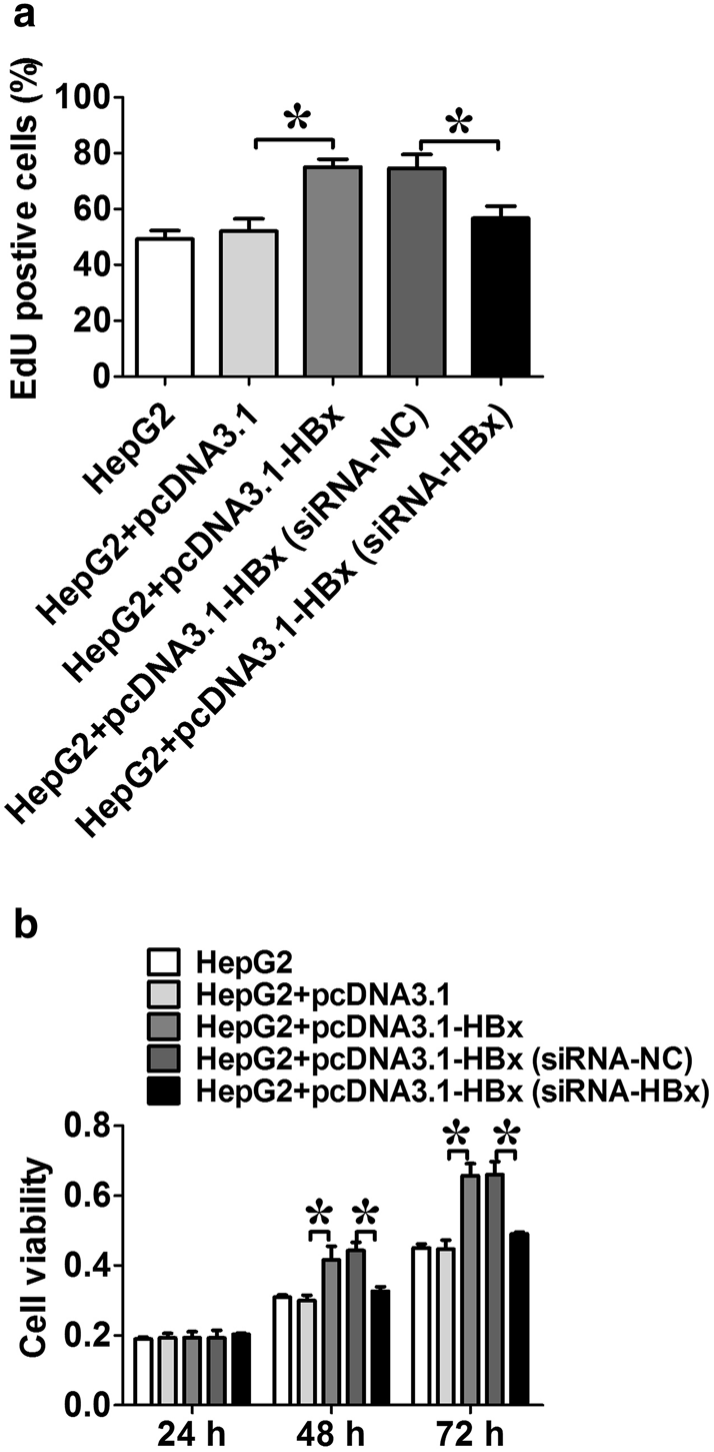
Effect of HBx on the cell proliferation in HepG2 cells. **a** The cell proliferation ability of hepatoma cells was analyzed using the EdU incorporation assay; **b** the cell proliferation ability of hetatoma cells was analyzed using MTT assay. *HBx* HBV X protein. Data represents the mean ± SEM, n = 3, *P < 0.05 (One-way ANOVA followed by Bonferroni test)

We also examined the effect of HBx on hepatoma cell proliferation. EdU incorporation assay and MTT assay results showed that HBx plasmid transfected HepG2 cells had a higher proliferation ability compared to HepG2 cells transfected with pcDNA3.1; and RNA interference targeting HBx mRNA abolished HBx-induced proliferation of HepG2 cells (Fig. 6, n = 3, *P* < 0.05).

### MiR-19a, miR-122 and miR-223 contribute to HBx-mediated proliferation of HepG2 cells

**Fig. 7:**
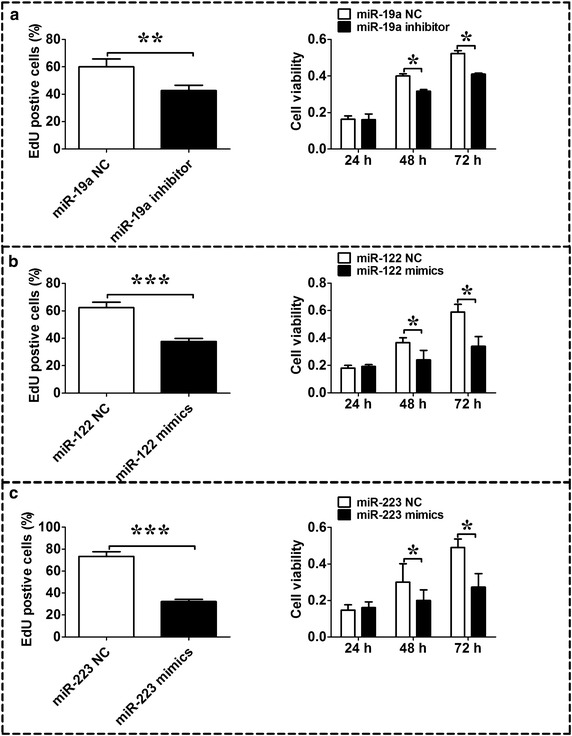
The role of miR-19a, miR-122, and miR-223 in HBx-mediated growth of HepG2 cells. **a** The proliferation ability of HepG2-pcDNA3.1-HBx cells was analyzed using the EdU incorporation and MTT assays after miR-19a inhibitor treatment; **b** the proliferation ability of HepG-pcDNA3.1 cells was analyzed using the EdU incorporation and MTT assays after miR-122 mimics treatment; **c** the proliferation ability of HepG-pcDNA3.1 cells was analyzed using the EdU incorporation and MTT assays after miR-223 mimics treatment. Data represents the mean ± SEM, n = 3, *P < 0.05; **P < 0.01; ***P < 0.001 (unpaired t-test)

The function of miR-19a, miR-122 and miR-223 in HBx-transfected HepG2 cells was also investigated. Previous results showed that miR-19a was up-regulated, miR-122 and miR-223 were down-regulated in HBx-transfected HepG2 cells. We elucidate the function of miR-19a by silencing the expression of miR-19a; and the function of miR-122 and miR-223 was determined by overexpression of miR-122 and miR-223. EdU incorporation assay and MTT assay results showed that silencing of miR-19a inhibited the growth of HBx-transfected HepG2 cells (Fig. 7a, n = 3, *P* < 0.05); the growth of HBx-transfected HepG2 cells was also inhibited by overexpression of miR-122 and miR-223, respectively (Fig. 7b, c, n = 3, *P* < 0.05).

### PTEN, cyclin G1, and c-myc contribute to HBx-mediated proliferation of HepG2 cells

**Fig. 8:**
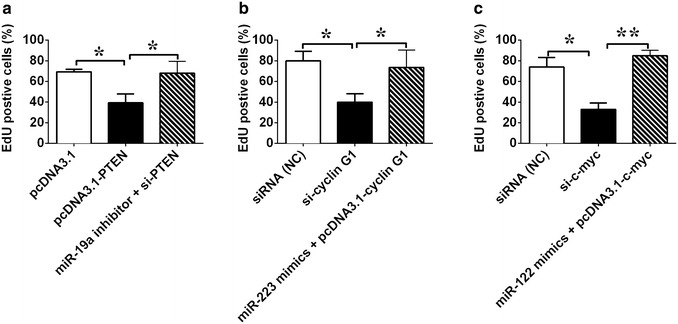
The role of PTEN, cyclin G1, and c-myc in HBx-mediated growth of HepG2 cells. The proliferation ability of HepG2-pcDNA3.1-HBx was analyzed using the EdU incorporation assays **a** after transfection with pcDNA3.1-PTEN or co-transfection with pcDNA3.1-PTEN and miR-19a inhibitor; **b** after transfection with cyclin G1 siRNA (si-cyclin G1) or co-transfection with pcDNA3.1-cyclin G1 and miR-223 mimics; **c** after transfection with c-myc siRNA (si-c-myc) or co-transfection with pcDNA3.1-c-myc and miR-122 mimics. Data represents the mean ± SEM, n = 3, *P < 0.05; **P < 0.01 (One-way ANOVA followed by Bonferroni test)

The function of PTEN, c-myc, and cyclin G1 in HBx- transfected HepG2 cells was further examined. EdU incorporation assay showed that transfection of PTEN expressing vector (pcDNA3.1-PTEN), cyclin G1 siRNA (si–cyclin G1) or c-myc siRNA (si–c-myc) inhibited the proliferation of HBx-transfected HepG2 cells (Fig. 8, n = 3, *P* < 0.05). Further rescue experiment showed that co-transfection with pcDNA3.1-PTEN and miR-19a inhibitor, pcDNA3.1-c-myc and miR-122 mimics or pcDNA3.1-cyclin G1 and miR-223 mimics restored the inhibitory effects (Fig. 8, n = 3, *P* < 0.05).

## Discussion

It is well known that HBx plays a key role in viral pathogenesis and hepatocarcinogenesis via modulation of cellular genes, which subsequently changes the cell signaling pathway and other cellular processes [3, 19, 20]. Enormous studies have shown that miRNAs involve in cell proliferation, tumorigenesis, apoptosis, invasion/metastasis and angiogenesis of cancer cells [8, 21]. In HBV-related HCC, miRNAs have been found to be involved in viral replication, latency, epigenetic modulation, interacting with viral products or indirectly alters cancer-related pathways via interacting with HBx [22]. In the present study, we first demonstrated that miR-19a, miR-122 and miR-223 were differentially modulated by HBx in HepG2 cells and HepG2.2.15 cells, and similar findings were further confirmed in the blood serum samples from HBV-positive HCC patients. Further studies also showed that modulated expression of miR-19a, miR-122 and miR-223 promoted cell proliferation of HBx-transfected HepG2 cells.

PTEN, cyclin G1 and c-myc, involving in cell proliferation, was found as targets for miR-19a, miR-122, and miR-223 respectively [11, 12, 16]. PTEN is known to be tumor suppressor that is mutated in a large number of cancers at high frequency [23]. It negatively regulates intracellular levels of phosphatidylinostitol-3,4,5-trisphosphate in cells and functions as a tumor suppressor by negatively regulating AKT/PKB signaling pathway [24]. MiR-19a has been shown to be an onco-miRNA by targeting PTEN in several studies [16, 25, 26]. In the present study, we found miR-19a was up-regulated in HBx–transfected HepG2 cells; and similar results were also found in HepG2 cells transfected with 1.3 fold HBV. The up-regulation of miR-19a in HBV-positive HCC patients in present study was in consistent with studies showing that miR-19a was both up-regulated in HCC tissues and blood serum from HCC patients [27, 28]. However, in HepG2.2.15 cells, we found the expression of miR-19a is lower compared to HepG2 cells, and its target PTEN mRNA and protein were up-regulated. We are still unclear of the underlying mechanism of this interesting finding. However, studies have shown that chronic HBV-induced HCC involves covalently closed circular episomal HBV genome as well as HBV DNA integrated host chromosome; trans-activation of HBx from covalently close circular episomal HBV genome may involve in cytoplasmic regulation of cell signaling pathway, which leads to hepatocarcinogenesis; while the HBV integration into host chromosome may play a role in modulation of genes that are responsible for cell proliferation [19, 29]. Therefore, we proposed that HBV employs both mechanism in HCC to differentially modulate miR-19a expression between HepG2.2.15 cells and HepG2 cells. Further studies may be required to clarify the underlying mechanism.

Cyclin G1, a target of miR-122, has been found to negatively regulate p53 protein stability by acting on PP2A, and its oncogenic role has been well documented in different human cancers [11, 30]. In the present study, miR-122 was found to be down-regulated in HBx-transfected HepG2 cells. Similar results were also observed in HepG2 cells transfected with 1.3 fold HBV genome and in HepG2.2.15 cells. In addition, miR-122 was also down-regulated in HBV-positive HCC patients [31]. Knock down of miR-122 experiment further confirmed that expression of miR-122 was dependent on HBx; and the regulation of HBx on miR-122 is possible via down-regulation of Gld2 [32].

C-myc is a regulator gene that codes for a transcription factor and plays a key role in cell cycle progression, apoptosis and cellular transformation [33], and overexpression of c-myc has been found in various types of cancers, including HCC [34, 35]. Present studies demonstrated that miR-223 is down-regulated in HBx or HBV transfected HepG2 cells and in HepG2.2.15 cells; and similar result was also found in clinically HBV-positive HCC patients, and our results from clinical samples were consistent with previous studies, which examined the expression of miRNAs in HBV-positive HCC patients. MiR-223 has also been shown to be tumor-suppressive in HCC [31], and repression of miR-223 has been associated with up-regulation of c-myc in HCC [12]. Wong et al., demonstrated that miRNA is commonly repressed in HCC and potentiates expression of Stathmin 1 [36], and further study also showed that miR-223 was epigenetically regulated by sulfatide and inhibits migration of HCC cells [37]. However, in certain types of cancers, miR-233 was found to be oncogenic. Notably, miR-233 is highly expressed in gastric cancer and promotes cell proliferation and invasion by targeting EPB41L3 [38]; and overexpression of miR-223 was found to correlate with tumor metastasis and poor prognosis in patients with colorectal cancer and plays an oncogenic role [39, 40]. Therefore, the role of miR-223 may be opposing in different types of cancer or tumor cell lines via different functional genes. In our study, the results showed the overexpression of c-myc mRNA and protein is associated with a down-regulation of miR-223 in HBx or 1.3 fold HBV-transfected HepG2 cells and in HepG2.2.15 cells, and up-regulation suppressed the cell growth in the HBx-transfected HCC, which may suggest that miR-233 is tumor-suppressive in HBV-related HCC.

Cell proliferation assay showed that HBx promoted cell proliferation of HepG2 cells, and this finding is consistent with previous studies [41]. We further confirmed that roles of miR-19a, miR-122 and miR-233 in cell proliferation in HBx-transfected HepG2 cells, with miR-19a being oncogenic, and miR-122 and miR-223 being tumor suppressive. MiR-19a has been found to promote cell proliferation in various types of cancers such as bladder cancer, gastric cancer, prostate cancer via targeting different transcriptional factors [16, 42, 43]; and in HCC patients, miR-19a was found to be up-regulated in the blood serum, and played inhibitory roles in HCC via regulating cyclin D1 expression [28], Melegari et al., further demonstrated miR-19a was up-regulated in HBV-positive HCC and this up-regulation is attributed to HBx [44]. In the present study, we further explored the role of miR-19a in HBV-related HCC and in three different HCC cell lines, and our results suggest that miR-19a is oncogenic in HBV-related HCC, and its oncogenic role may be via regulating PTEN regulation, although the underlying mechanism of miR-19a involved in hepatocytes cell proliferation needs further investigation. MiR-122 has been found to be tumor suppressive in liver cancer, dysregulation of miR-122a has been found in HBV-related HCC and liver cirrhosis [31, 45]. The role of miR-122 in cell proliferation may involve up-regulation of PTTG-1 binding protein [46] and cyclin G1 [11] in HBx-transfected HepG2 cells. Down-regulation of miR-223 has been verified in several HCC studies [47, 48] and its down-regulation is associated with the epigenetic regulation by highly expressed sulfatide and involved in tumor metastasis; and the regulatory role of miR-223 in hepatocarcinogenesis may involve modulation of several targets including integrin αV and c-myc [12, 37]. Our rescue experiments further confirmed that these miRs regulated cell proliferation via targeting their respective targets, i.e. PTEN, cyclin G1 and c-myc. Collectively, our results demonstrated that dysregulation of miR-19a, miR-122 and miR-223 in sera may be associated with the pathogenesis of HBV-related HCC, which may suggest that serum miR-19a, miR-122 and miR223 might serve as a novel and potential noninvasive biomarker for prognosis of HBV-related HCC; and modulation of these miRs expression may provide novel therapeutics for the treatment of HCC. However, the underlying mechanisms of miR-19a, miR-122 and miR-223, and their respective targets involving in cell proliferation in HBx-transfected HepG2 still need further investigation.

## Conclusions

In conclusion, present studies demonstrate that HBx protein differentially regulated the expression of miR-19a, miR-122 and miR-223, and its regulation may be associated with genotype C; miR-19a, miR-122 and miR-223 might also involve in the hepatocarcinogenesis in HBV-related HCC. Our results may provide new insight into understanding the interaction between HBx and miRNAs and their regulatory roles in HBV-related HCC.

## Abbreviations

EdU: 5-ethynyl-2-deoxyuridine
HBV: hepatitis B virus
HBx: HBV X protein
HCC: hepatocellular carcinoma
miRNA: microRNA
qRT-PCR: quantitative real time PCR
